# Microfluidic-based processors and circuits design

**DOI:** 10.1038/s41598-021-90485-z

**Published:** 2021-05-26

**Authors:** Kasra Azizbeigi, Maysam Zamani Pedram, Amir Sanati-Nezhad

**Affiliations:** 1 Faculty of Electrical Engineering, K.N. Toosi University of Technology, Tehran, Iran; 2 Department of Mechanical and Manufacturing Engineering, University of Calgary, Calgary, AB T2N 1N4 Canada; 3 Biomedical Engineering Program, Center for Bioengineering Research and Education, University of Calgary, Calgary, AB T2N 1N4 Canada

**Keywords:** Fluid dynamics, Engineering

## Abstract

Droplets produced within microfluidics have not only attracted the attention of researchers to develop complex biological, industrial and clinical testing systems but also played a role as a bit of data. The flow of droplets within a network of microfluidic channels by stimulation of their movements, trajectories, and interaction timing, can provide an opportunity for preparation of complex and logical microfluidic circuits. Such mechanical-based circuits open up avenues to mimic the logic of electrical circuits within microfluidics. Recently, simple microfluidic-based logical elements such as AND, OR, and NOT gates have been experimentally developed and tested to model basic logic conditions in laboratory settings. In this work, we develop new microfluidic networks, control the shape of channels and speed of droplet movement, and regulate the size of bubbles in order to extend the logical elements to six new logic gates, including AND/OR type 1, AND/OR type 2, NOT type 1, NOT type 2, Flip-Flop, Synchronizer, and a parametric model of T-junction as a bubble generator. We further designed and simulated a novel microfluidic Decoder 1 to 2, a Decoder 2 to 4, and a microfluidic circuit that combines several individual logic gates into one complex circuit. Further fabrication and experimental testing of these newly introduced logic gates within microfluidics enable implementing complex circuits in high-throughput microfluidic platforms for tissue engineering, drug testing and development, and chemical synthesis and process design.

## Introduction

The innovations in microfluidic Lab-on-chip (LOC) technologies have exploited the advantages of micro-scale manipulation, merging and mixing of particles for mechanization of numerous phenomena in industries, biological systems and clinics^1^. The advances in droplet microfluidic platforms that are based on two-phase flow systems and denomination of bubble logics have enabled on-chip microfluidic data streaming^2,3^. In the two-phase flow systems with two immiscible fluids transporting through microchannels^4–6^, the initial module conveying data requires bubbles (droplets). Instead of utilizing a high or low electrical voltage, the existence or non-existence of a bubble is defined as a bit of data. The heuristic design of microfluidic chips that employ the interplay of bubbles streaming through microchannels does not usually require complex control valves and active switching equipment.

To implement logic operations in microfluidic systems, knowledge in fluidic mechanics and dynamics of multiphase flows is required^7,8^. Liquid-based logic systems are not the same as solid-state systems according to a computational power. Instead, they offer an effective method to execute an independent passive control in microfluidic frameworks. The parameters normally used to passively control the bubbles are flow resistance and viscosity of the phases which resemble a memory, while the shape and connectivity of microchannels enable diverse microfluidic logic gates^9–11^. A reliable method to predict the behavior of fluidic logic gates is the analysis of two-phase flow systems which helps to execute universal Boolean operators and provides the opportunity to design droplet-level mechanisms in microfluidic operators^12^.

The AND/OR/NOT gates, Flip-Flop, Synchronizer, and electro-bubble modulator have been demonstrated in a set of microfluidic chips to execute logic operations^2^. For example, two models of AND/OR and NOT gates^13^ and models of AND/OR gate, NOT, Flip-Flop, and T-junction^14^ have been simulated within microfluidics. However, all these models rely on suppositions applied to the channels and fluids^15^. These models though deal with highly nonlinear parameters which require further consideration for simulating complex circuits. Examples of these nonlinearities are related to changes in dynamic viscosity, hydraulic resistance, and consistency variation at the location of bubbles^16^.

In this study, eight new circuit models relevant to six different microfluidic chips were simulated. The fluidic dynamics in accordance with the behavior of the electrical logic gates were analyzed, and various bubble logic circuits, including AND/OR logic gate 1, AND/OR logic gate 2, logic NOT gate type 1, logic NOT gate type 2, Flip-Flop memory gate, Synchronizer, logic Decoder 1 to 2 gate, and logic decoder 2 to 4 gate were modeled^2,13^. Similar to a voltage generator in electric circuits, a microchannel model was designed as a bubble generator to control flow rate at input gates^17^, wherein the comparison between the computational models and experimental results were accomplished^18,19^. The high efficiency of the individual logic gates was demonstrated and used to introduce a modular combinational logic with an appropriate efficiency. Exclusively, in the T-junction model, practiced as a bubble generator, the performance of the executed model was evaluated considering the diversity of flow rates, channels geometry, and grid-independence. The proposed microchannel designs are tentative to present the multifaceted nature of the multiphysics marvels in microfluidic systems^20,21^. Although there are other pairs of fluids in droplet microfluidics other than the water (e.g. water-hexadecane)^14^, here we focused on the interaction analysis of the two incompatible fluids of the water and the air^22,23^. The success of developing various simple and complex circuit models in the middle- or high-throughput platforms by a combination of circuit elements developed in this work enables the automation of complex reaction, interaction, and sensing assays used in high-throughput droplet microfluidics (biofuel generation, tissue engineering, drug testing, production of smart composite particles, emulsions and microgels)^24^, digital microfluidics (chemical and biochemical reactions, biosensing assays, single-cell analysis)^25–29^, thermofluids (point-of-care devices, in-vitro diagnostics, environmental sensing)^30^, and optofluidics (bioanalysis)^31^, with the applications in chemistry, energy, biology, medicine, and environment.

## Results

### T-Junction microfluidic structure

**Figure 1:**
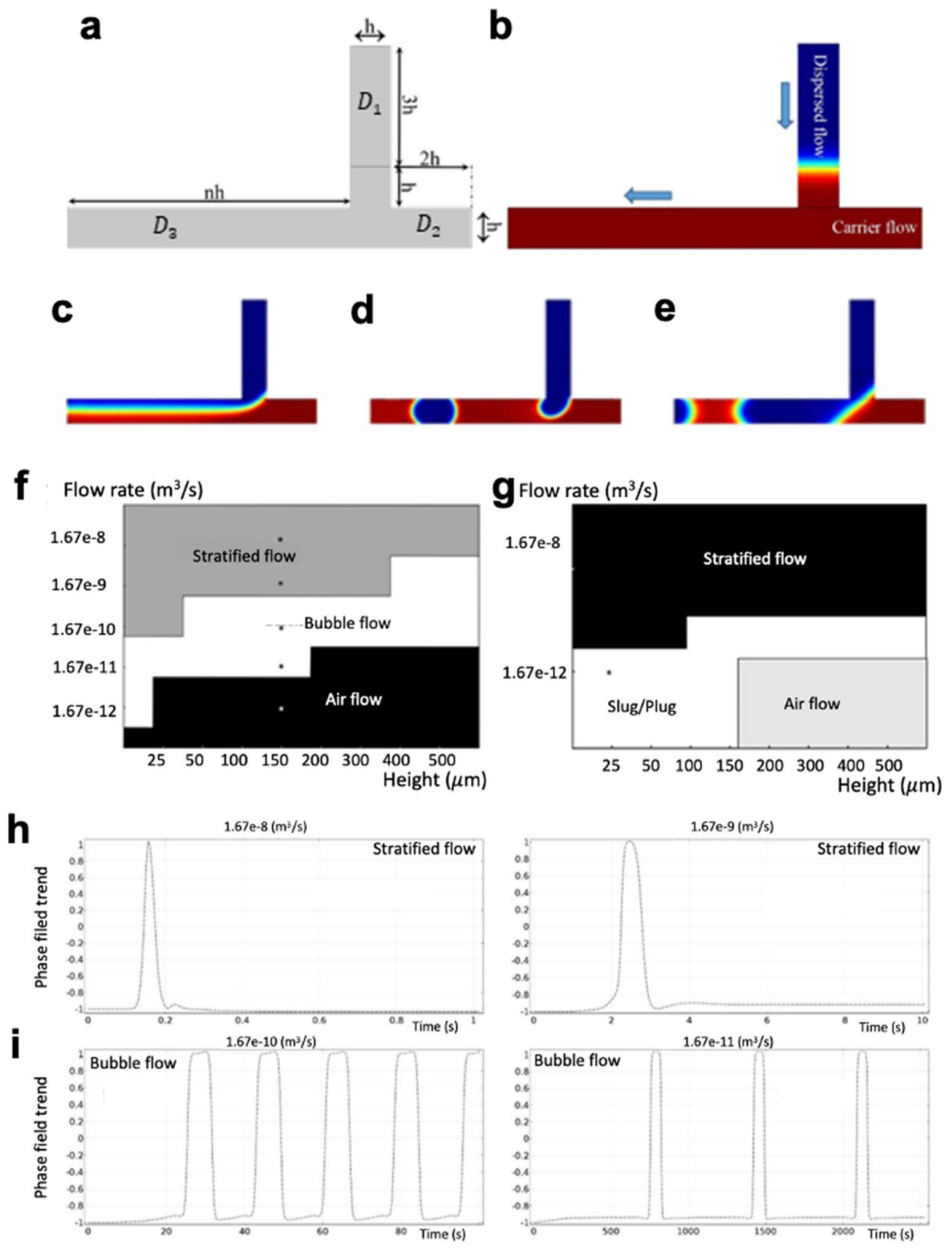
The T-junction microfluidic model and its function for the formation of different flow regimes. (**a**) The geometry design of the T-junction structure where each part scaled in the order of the channel height (h). (**b**) The two liquid phases in the T junction model, including carrier phase (air) in domains *D2* and *D3*, and the dispersed phase (water) in domain *D1*. The difference in flow patterns is based on the flow rate ratio of the two fluids, resulted in the formation of (**c**) Stratified flow, (**d**) Bubble flow, and (**e**) Slug/Plug flow. (**f,g**) The phase plot of the water–air droplet generation model for different flows and heights of the microchannels (Mesh degree: Normal). We tagged some points in the phase plots to investigate the phase-field trends. (**h**) The phase field approach versus time in Stratified flow, wherein flow rate of the carrier fluid ($${Q}_{c})$$ = flow rate of the disperse fluid ($${Q}_{d}$$), the initial entrance length in the carrier fluid channel $${D}_{{z}_{c}}=40 \mu m,$$ and the initial entrance length in the disperse fluid channel $${D}_{{z}_{d}}=70 \mu m$$. (**i**) $${Q}_{c} = 10 * {Q}_{d}$$ ($${D}_{{z}_{c}} = 40 \mu m, {D}_{{z}_{d}} = 70 \mu m$$).

A T-junctions model was designed and used as a standard bubble generator (for all logic gates studied in this work). The T junction model generates bubbles crossing into a channel while controlling the flow rate at the gate inputs. The carrier and dispersed phases are infused into the microfluidic chip by two different inlets. The dispersed phase (the water) is slowly infused from the first inlet into the main channel where the carrier phase (the air) enters from the second inlet. The shear stress and the design of the microchannels contribute to the formation of bubbles. The intersection point forces the bubbles to form with their size dependent on the ratio of the input flow rate. For the T-junction model, the channel network was separated into three main domains (Fig. 1a). The domain *D*_*1*_ was loaded with the dispersed fluid (Fig. 1b, blue section) while the carrier fluid (Fig. 1b red section) was loaded with the domains *D*_*2*_ and *D*_*3*_. The boundary conditions of the microchannels were set to the no-slip condition for the dispersed phase in *D*_*1*_ and the wetted wall condition for the carrier phase in *D*_*2*_ and *D*_*3*_. The laminar flow ratio of the two inlets controls the velocity of the dispersed fluid in sections *D*_*1*_ and *D*_*3*_ (based on the continuity and Navier–stokes equations). The outlet pressure in *D*_*3*_ was set to zero. The interior boundary was set as an initial fluid interface between the sections to separate the dispersed phase from the carrier fluid at domains *D*_*1*_ and *D*_*2*_. The interior wall between sections *D*_*2*_ and *D*_*3*_ was set to a continuity wall. Figure 1c–e shows the conditions in which the Stratified flow (parallel flow of two or more liquids along the channel), Bubble flow (a multiphase flow wherein droplets of one phase are well-formed), and Slug/Plug flow (a multiphase flow wherein ‘bullet-shaped’ droplets are formed and cover the cross-section of the channel) are created, respectively. Figure 1f,g shows the effect of channel height as well as flow rates of the carrier and disperse phases (Q_c_ and Q_d_, respectively) on forming liquid flow patterns and shapes of bubbles. Figure 1h,i shows the phase-field trend (see details in Supplementary Information) versus time for three different flow regimes created in Fig. 1c–e. It is noted that the experimental design of the T-junction would not only contains the main microchannel network shown in Fig. 1a but also the large and wide channels connecting the inlets and outlet reservoirs to the main microchannel network shown in Fig. 1a.

The input flow rate for all the logic gates was modeled such that the amplitude of the flow rate was set to zero at $t=0$ and gradually increased to a given input flow rate at t_1_ = 1. Given the costly three-dimensional (3D) simulation of the logic gates^32^, we developed 2D symmetric models with the flow dynamics fixed along the third dimension to reduce the complexity of the numerical simulation. The time step of the simulation varied between 0.01 ms to 1 ms $.$ To simulate a valid logic model of the T-junction, the effects of several parameters including channel height, flow rate ratio, meshing parameters, and contact angles were examined (Supplementary Information S1). It is noted that the flow of smaller bubbles in longer channels resulted in the phase-field values between − 0.8 and + 0.8. This fluctuation in the phase-field trend is detectable in simulation data (S1).

### The microfluidic AND/OR type 1 gate circuit

**Figure 2:**
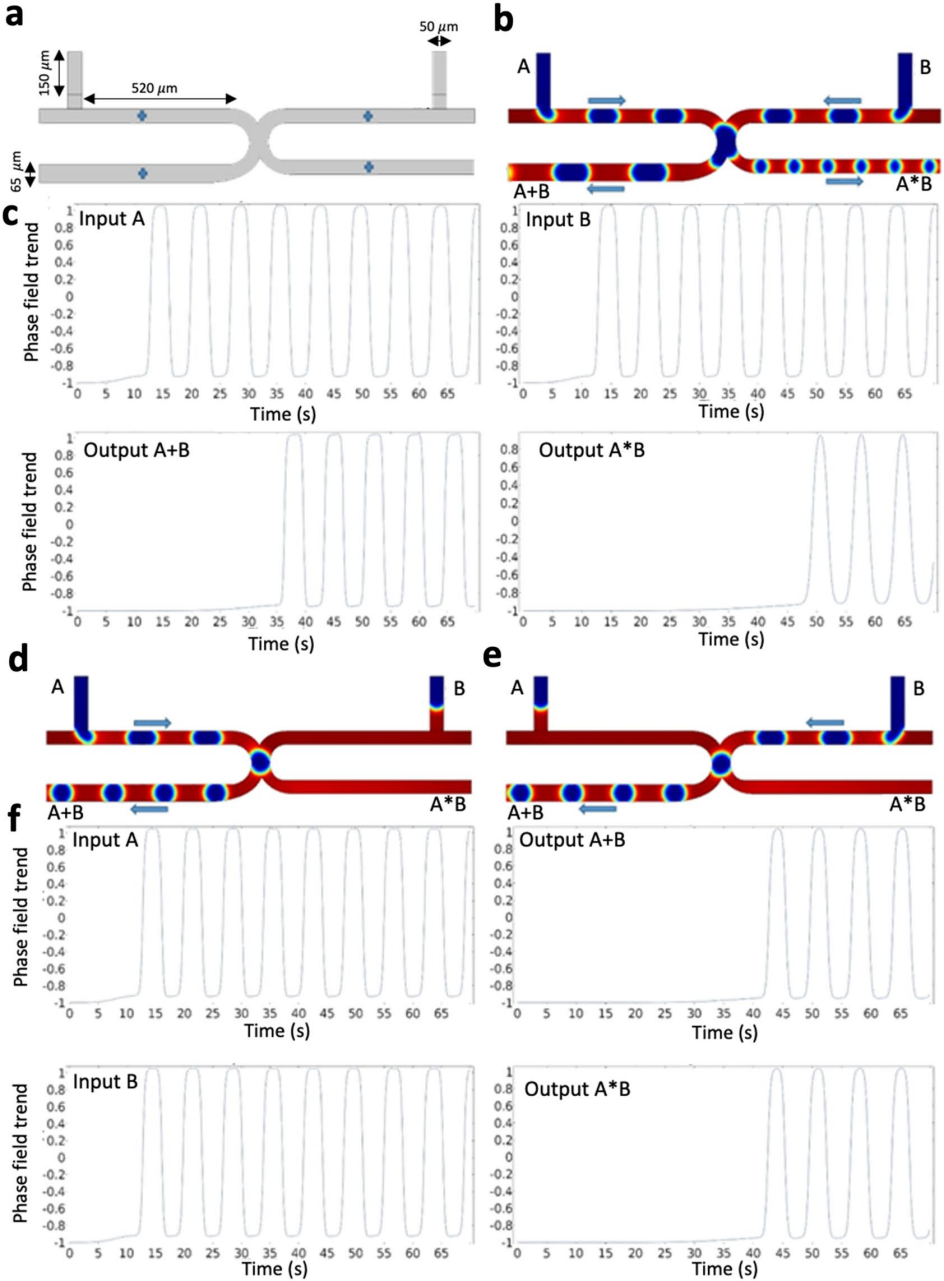
The microfluidic AND/OR type 1 gate. (**a**) The design and geometry of the microchannels. Probe points (blue points) are used to quantify the phase-field value. (**b**) A snapshot in the case (1–1) and an indication of the bubble direction. (**c**) Phase-field trends of both the inputs (having the same trend), and phase field trends of the outputs ($A+B$) and ($A*B$). The difference between the phase-field trend of ($A+B$) and ($A*B$) shows that the bubbles in the OR gate reach the output sooner than the AND gate. (**d**) A snapshot in the case (1–0), and (**e**) a snapshot in the case (0–1). (**f**) The phase-field trends of the input A and the output ($A+B$), and the phase-field trend of the input B and the output ($A*B$).

In this gate model, the geometry was designed based on two T-junctions^2^. Figure 2a shows the fluid flow behavior of the AND/OR logic gate. Starting from inputs $A$ and $B$, a synchronous train of bubbles is generated and conducted through the channels. The output branches are tagged as $A+B$ for the OR gate and $A*B$ for the AND gate. There are four logic conditions in this scenario. In the case $A=0$ and $B=0$, no bubble is produced at the outputs ($A+B=0$, $A*B=0$) (Fig. 2a). In the case $A=1$ and $B=1$, the resulted outputs are $A+B=1$ and $A*B=1$ (Fig. 2b,c). In the cases $A=1$, $B=0$ or $A=0$, $B=1$, the resulted outputs are $A+B=1$ and $A*B=0$ (Fig. 2d–f) [Videos S1, S2, and S3].

### The microfluidic AND/OR type 2 gate circuit

**Figure 3:**
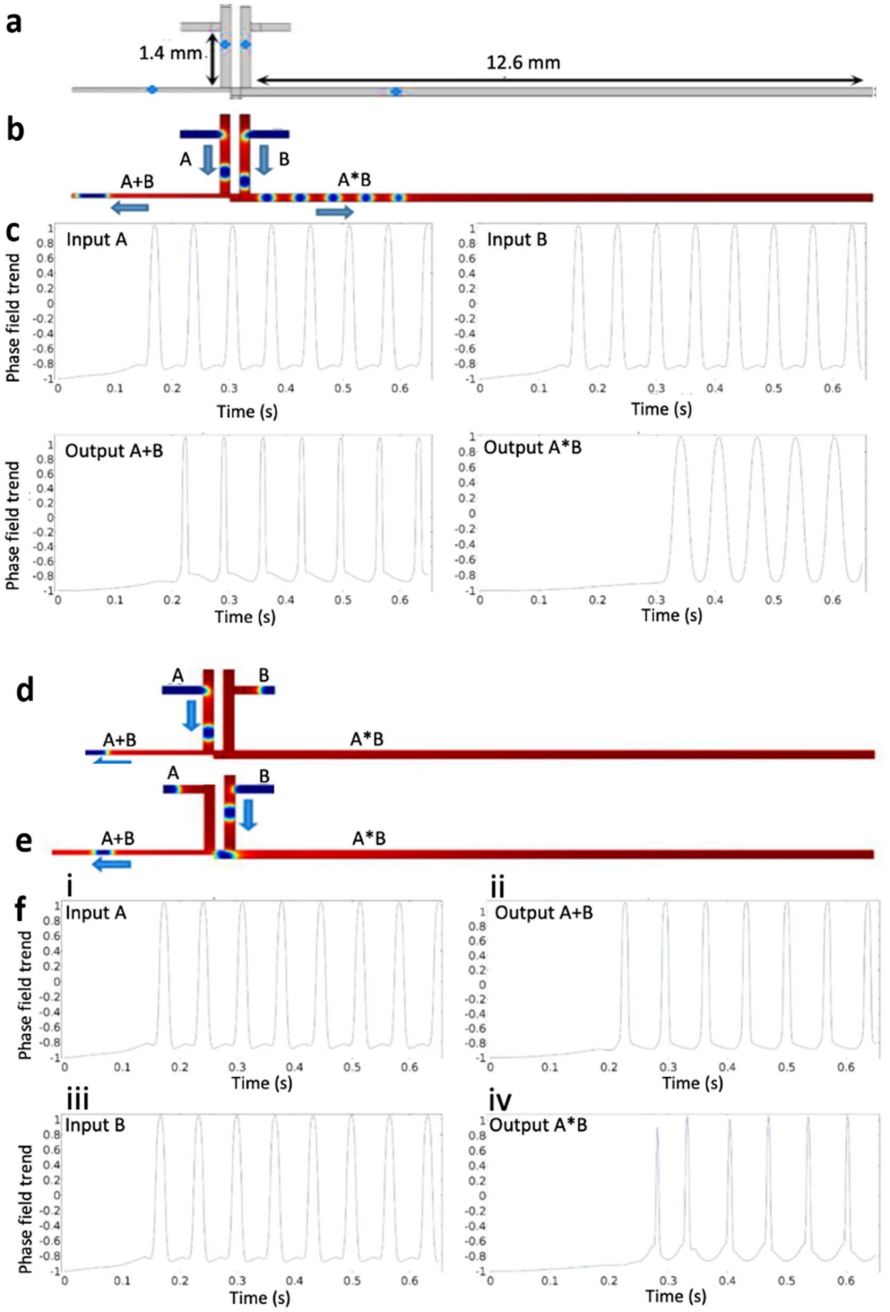
The microfluidic AND/OR gate type 2. (**a**) The design and geometry of the microchannels, and the probe points wherein the phase-field trends are quantified. (**b**) A snapshot in the case (1–1) and an indication of bubbles direction. (**c**) Phase-field trends of both inputs (both inputs have the same trend), output ($A+B$), and output ($A*B$). The differences between phase-field trends of output ($A+B$) and output ($A*B$) show that the bubbles in the OR gate reach the output faster than the AND gate. Moreover, due to the hydraulic resistance of the OR output branch, the phase-field trends in the top and bottom points are more compact. However, it does not make any significant difference in the path of droplets where each droplet shows a bit of data equals to 1. (**d**) A snapshot in the case (1–0) and an indication of bubbles direction. (**e**) A snapshot in the case (0–1) and an indication of bubbles direction. (**f**) The phase-field trends of input $A$ and output ($A+B$), and the phase-field trends of input $B$ and output ($A+B$). The difference between the cases (1–0) and (0–1) due to the differences in their hydraulic resistance. The incomplete peaks with narrow bands seen in **(f)**. (iv) In outlet channel A*B are artifacts (not real peaks) in the numerical simulation, as demonstrated in Video S4. Therefore, no droplet is formed in channel outlet A*B while all droplets are conducted to the outlet channel A + B.

The structure of the AND/OR type 2 gate microfluidic circuit is designed with two T-junctions (Fig. 3a)^13^. A symmetric structure with an excluded bending shape of the AND/OR type 1 design is the main improvement compared to the type 1 system. Moreover, there is no interface between the bubbles produced from two different generators in AND/OR type 2 gate circuit. The bubbles are introduced from two different inputs and select their path based on the hydraulic resistance of the microchannels. The right branch that is longer and broader than the left branch is tagged with $A*B$ for the AND gate and has a higher hydraulic resistance than the OR gate tagged as $A+B$. It is essential to select appropriate input flow rates and the entrance length for each of the phases to warrant the formation of the bubbles at the output microchannels. Similar to the AND/OR type 1 gate, there are four flow scenarios in this AND/OR type 2 gate. In the logic condition (0–0) where no bubble is generated, the input gates are set to $A=0, B=0$ wherein the outputs are $A+B=0$ and $A*B=0$. Where both bubble generators produce bubbles, the input gates are set as $A=1, B=1$. Because of the presence of the bubbles in the OR gate that increases the hydrodynamic resistance in this gate compared to the AND gate, the outputs are $A+B=1, A*B=0$ (Fig. 3b,c). In the logic condition (1–0) or (0–1) where only one of the bubble generators produce bubbles, the input gates are set as $A=1, B=0$ or $A=0, B=1$. Because of the higher hydrodynamic resistance of the AND gate, the outputs are $A+B=1$ and $A*B=0$ (Fig. 3d–f). The hydraulic resistance $R=\mu \times \frac{h}{\omega }$ is proportional to dynamic viscosity ($\mu$) and channel height ($h$), and inversely proportional to channel width ($\omega$)^15^. The experimental results of Cheow et al.^13^ verify our 2D numerical model for this gate circuit.

**Figure 4:**
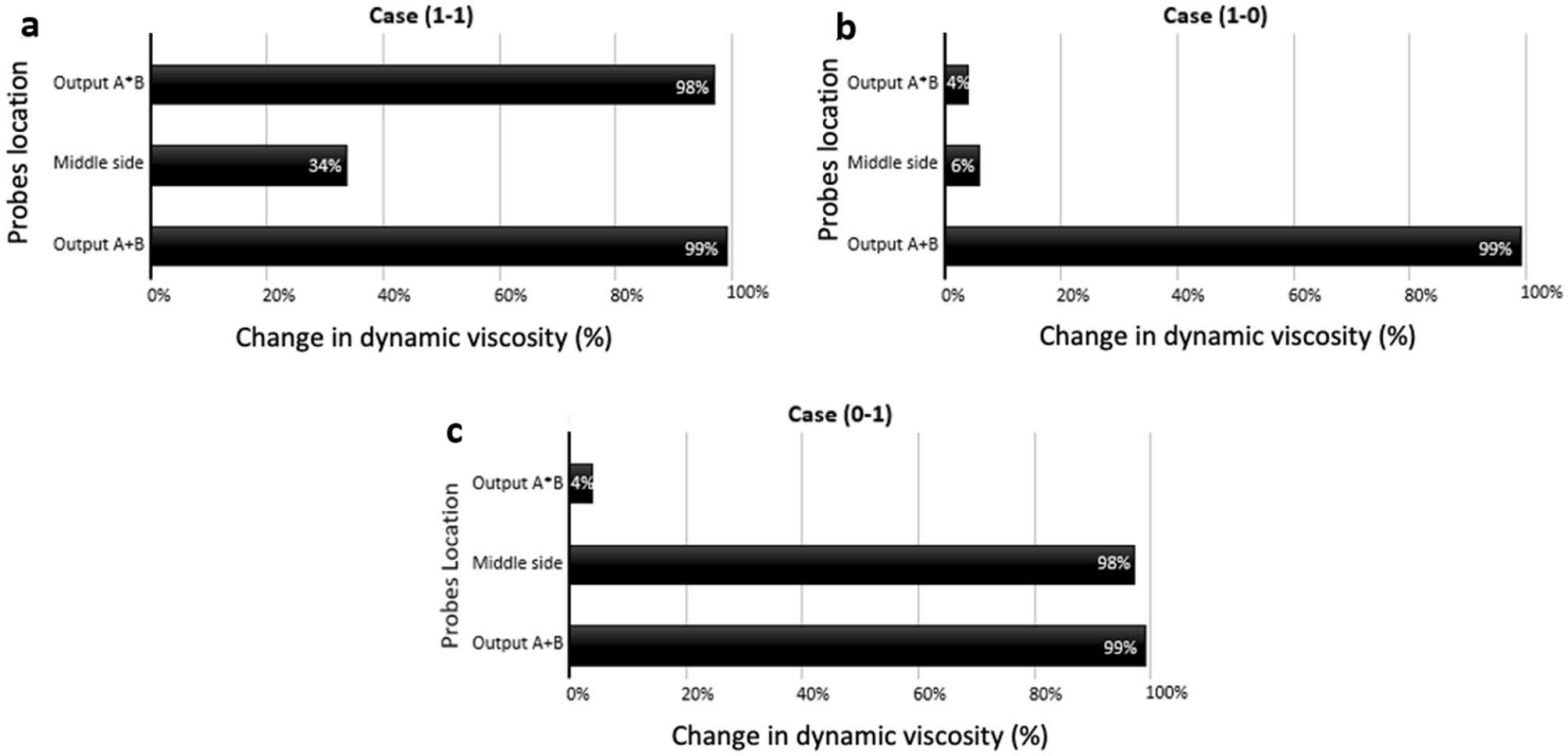
The change in dynamic viscosity of the bubble/liquid for the logic AND-OR type 2 gate circuit. The percentage change of dynamic viscosity of the bubble/fluid at three different points of the microfluidic network: Output $A+B$, Middle side, Output $A*B$. (**a**) Case (1–1), (**b**) Case (1–0), (**c**) Case (0–1). In all graphs, the percentage change of dynamic viscosity for the OR output branch is almost equal to 100%. In the logic condition (1–1), the percentage change of dynamic viscosity is almost 35% because the bubbles may choose their path to the other output branch.

The dynamic viscosity is calculated in three points of the output channels: in the OR branch, in the AND branch, and in the middle of the microchannel between the two input gates. The percentage of changes in dynamic viscosity is plotted in Fig. 4, which is mainly influenced by the presence or absence of the bubbles in the microchannels (Videos S4, S5 and S6).

### NOT type 1 microfluidic circuit

**Figure 5:**
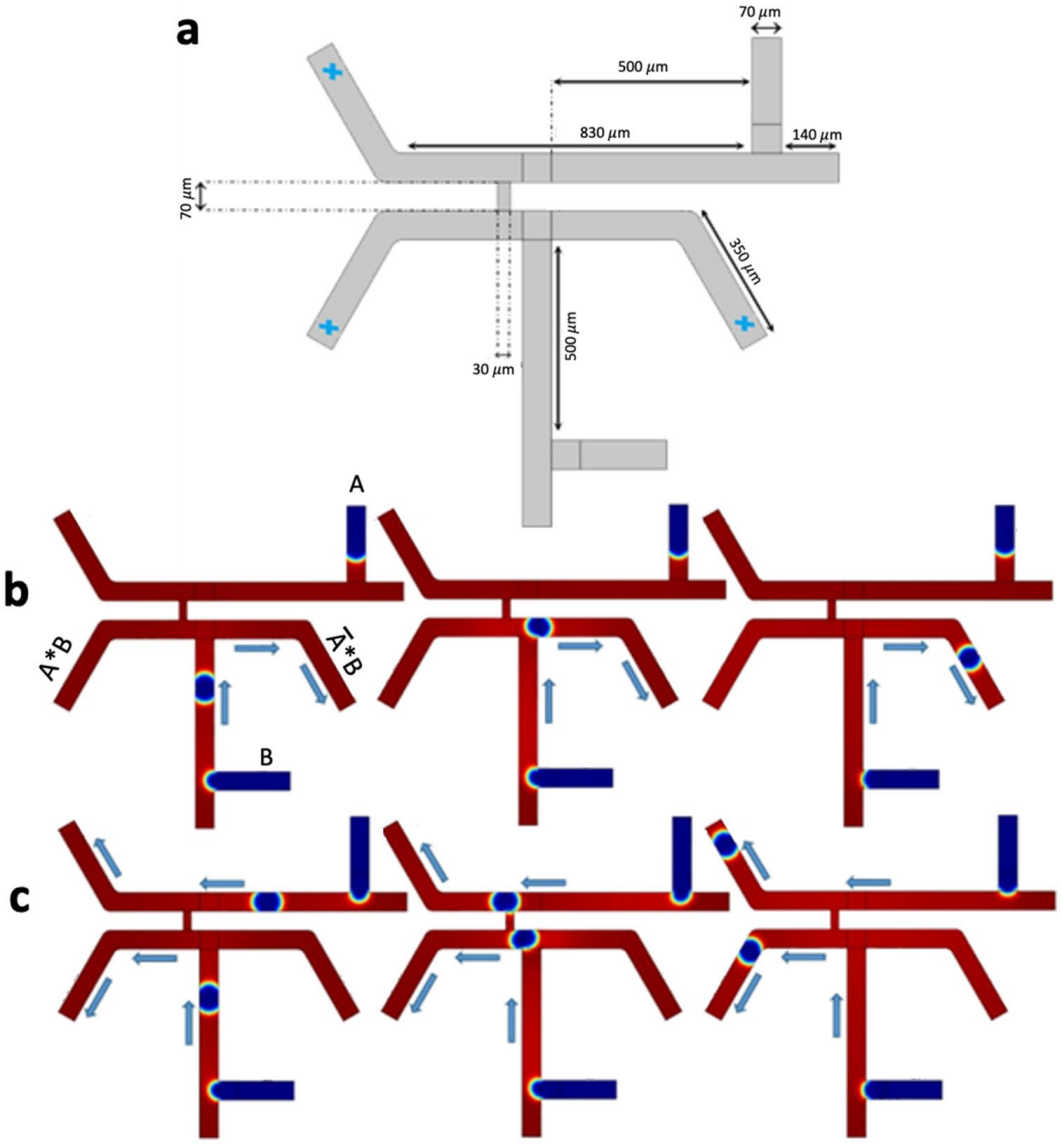
NOT gate type 1 circuit. (**a**) The design and geometry of the fluidic network for NOT gate type 1. (**b**) Series of snapshots for case $A=0, B=1$, wherein the direction of bubble flow is toward output $\overline{A }* B$. (**c**) Series of snapshots for case $A=1, B=1$, wherein the direction of bubble flow toward outputs $A$ and A*B.

For NOT gate type 1 microfluidic circuit, the geometry of the microchannel network contains two T-junctions for bubble generation (channel height: 100 µm) (Fig. 5a). There are two inputs at the middle and right sides of the microfluid network (inlets $A$ and $B$) which produce the bubbles. There are three output gates, one at the top left and one at the bottom right ($D1$ and $D2$) used as a drain, and the third one as the NOT gate ($\overline{A }$). The output branches are named as $A$, $\overline{A }*B$, and $A * B$. Input A plays a role as a buffer to show whether the desired gate produces a bubble. The NOT gate type 1 has two different functions. In the case $A=0$ and $B=1$, the upper input gate does not produce bubble and show an increased hydraulic resistance of the left side of the microchannel. Therefore, the bubbles produced from input *B* flow through gate $\overline{A }*B$ (Fig. 5b). In the case $A=1$ and $B=1$, by producing bubbles at the upper input gate, the hydraulic resistance of the right side of the microchannel increases and bubbles produced from input *B* flow through the output branch $A * B$ **(**Fig. 5c). The narrow path interconnecting the upper and lower branches is the main hydraulic resistance in the fluidic network, controlling the performance of the NOT logic gate (Video S7 and S8).

### NOT type 2 microfluidic gate circuit

**Figure 6:**
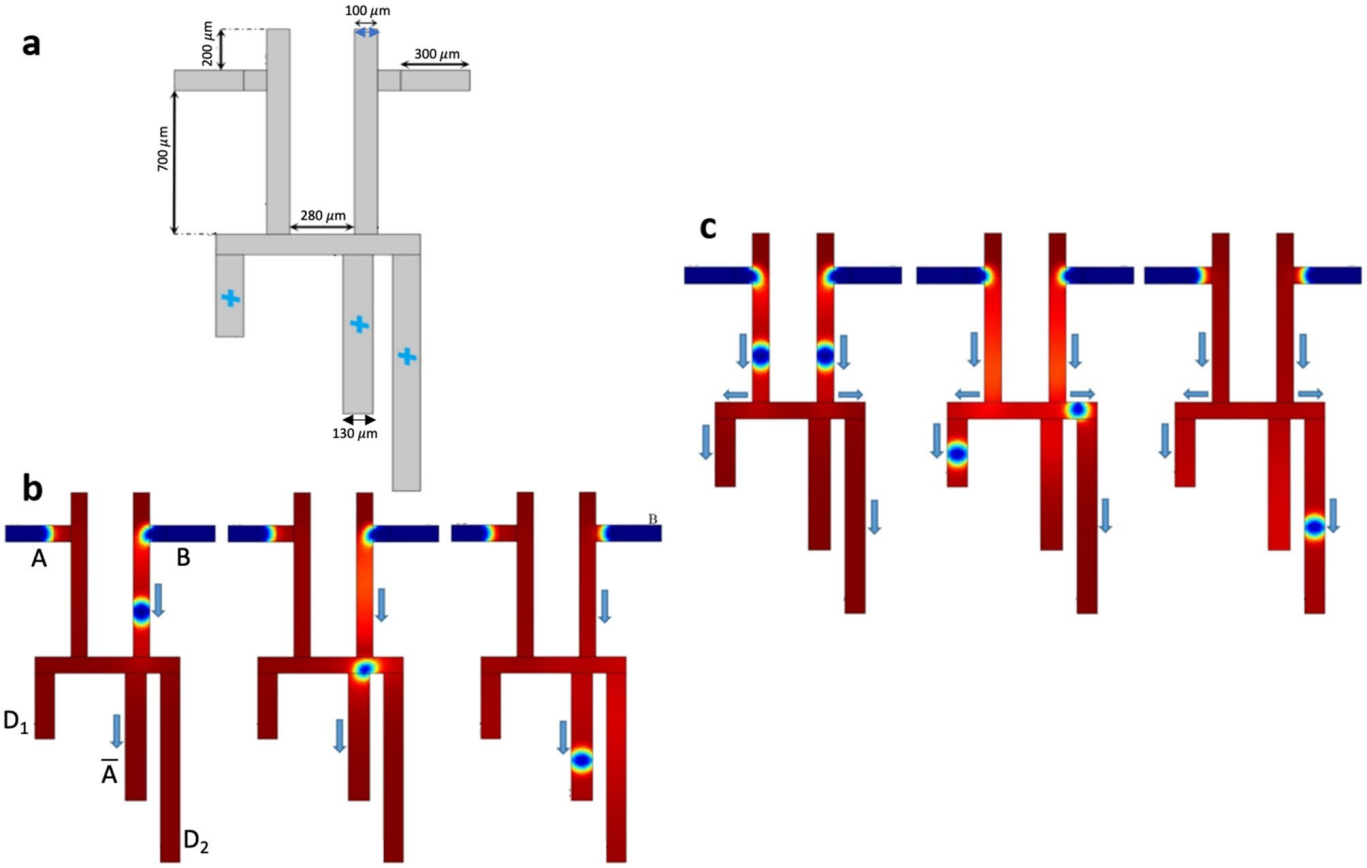
The microfluidic design of NOT gate type 2. (**a**) The design and geometry of NOT gate type 2 and the probe points at the outlets. (**b**) Series of snapshots for the logic condition $A=0, B=1$, wherein the bubble flow is toward output $\overline{A }$. (**c**) Series of snapshots for the logic condition $A=1, B=$ 1, wherein the bubble flow is toward outputs ${D}_{1}$ and ${D}_{2}$.

The design and geometry of NOT gate type 2 circuit contain two T-junctions for bubble generation (channel height: 100 µm) (Fig. 6a). Two inputs were placed vertically at the top of the network (Inputs $A$ and $B$) to produce a single bubble. The two output gates placed vertically at the bottom of the fluidic network ($D1$ and $D2$) function as a drain. The other output branch plays the role of NOT gate ($\overline{A }$). The movement of bubbles through the network is controlled by adjusting the flow rate of the inputs enabling the function of NOT gate type 2. In the case $A=0, B=1$, there is no droplet formed in $A=0$, and therefore it is vital to preserve the hydraulic resistance equilibrium such that input $B$ always produces bubbles. In this scenario, without producing any bubble via input $A$, the bubbles produced from input B flow through gate $\overline{A }$ (Fig. 6b). For the input condition $A=1, B=1$, both the inputs produce bubbles to equalize the hydraulic resistance in the two side channels of the fluidic network (right and left), leading to bubble flow into the gates $D1$ and $D2$ (Fig. 6c). The narrow path between these two side channels is the primary mediator of the fluidic network’s resistance.

**Figure 7:**
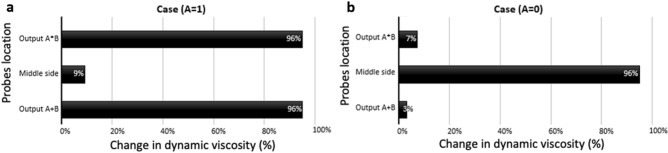
The percentage change of dynamic viscosity for the three selected points at the outlets in the fluidic network: 1. Output $D1$, 2. Output $\overline{A }$, and 3. Output *D*_*2*_. (**a**) The case $A=0$, (**b**) The case $A=1$. In the case of $A=0$, where bubbles reach output $\overline{A }$, the percentage change of dynamic viscosity is almost 100%. In contrast, in the case $A=1$, the percentage change of dynamic viscosity in $\overline{A }$ branch is almost zero.

To validate our computational model, the variation in dynamic viscosity was calculated at three selected points at the output branches in the fluidic network. Figure 7 shows the change in dynamic viscosity for these three points $\overline{A }$, and $D1$ and $D2$ branches [Videos S9 and S10].

### The microfluidic flip-flop gate

**Figure 8:**
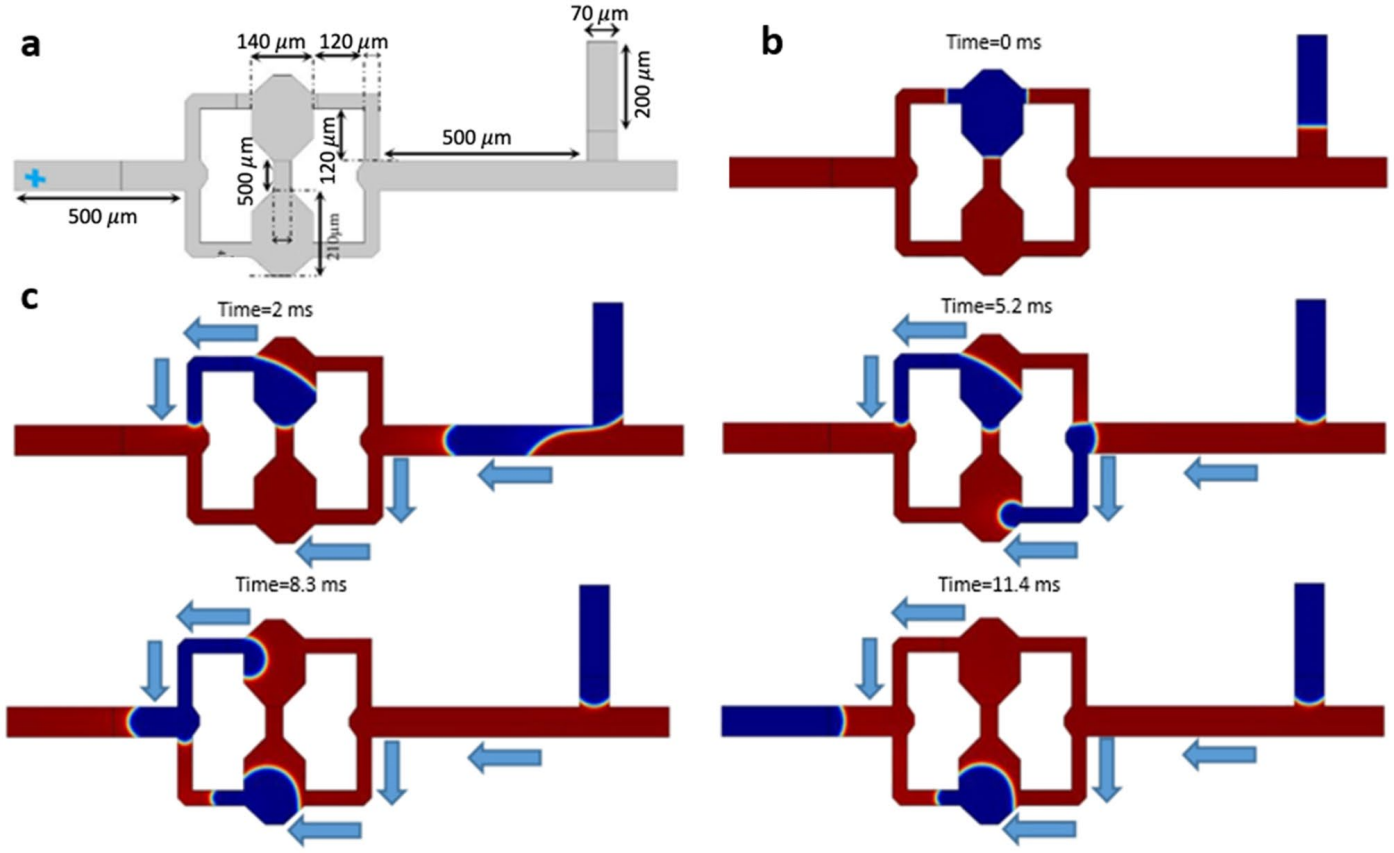
The microfluidic-based design of the Flip-Flop gate. (**a**) The design and geometry of the Flip-Flop gate. (**b**) A snapshot at t = 0. (**c**) Series of snapshots of the function of the Flip-Flop gate from t = 2 ms to t = 11.4 ms, and the sequence of bubble formation and direction. Activating one of the upper or lower cells in the Flip-Flop chip (by storing one bit of data (droplet)) represents the function of a microfluidic Flip-Flop gate as a memory circuit.

The microfluidic Flip-Flop gate model as a memory circuit has two T-junctions for bubble generation (channel height: 70 µm) (Fig. 8a). This model uses an unstable function by toggling off the bubbles, meaning that the fluidic network holds a single bubble by default until the other bubble arrives from the input branch and makes it toggle. The flow parameters and channel geometries need to be accurately selected to warrant synchronizing the processes of generating the first bubble and arrival of the second bubble. The period between the generation of these two bubbles is larger than the average time taking a bubble reaches output (*t*_*out*_). As shown in Fig. 8b, when the first bubble is stored at the upper cell of the fluidic network at $t=0$, the other bubble was not yet produced. Upon the generation of the second bubble (Fig. 8c), the change in hydraulic resistance of the fluidic network enables the initiation of the flow of the stored bubble at the upper cell. However, due to the presence of the first bubble in the upper cell, the second bubble is trapped and stored at the lower cell. This process consecutively continues, confirming the function of a microfluidic Flip-Flop circuit (Video S11).

### The microfluidic synchronizer gate

**Figure 9:**
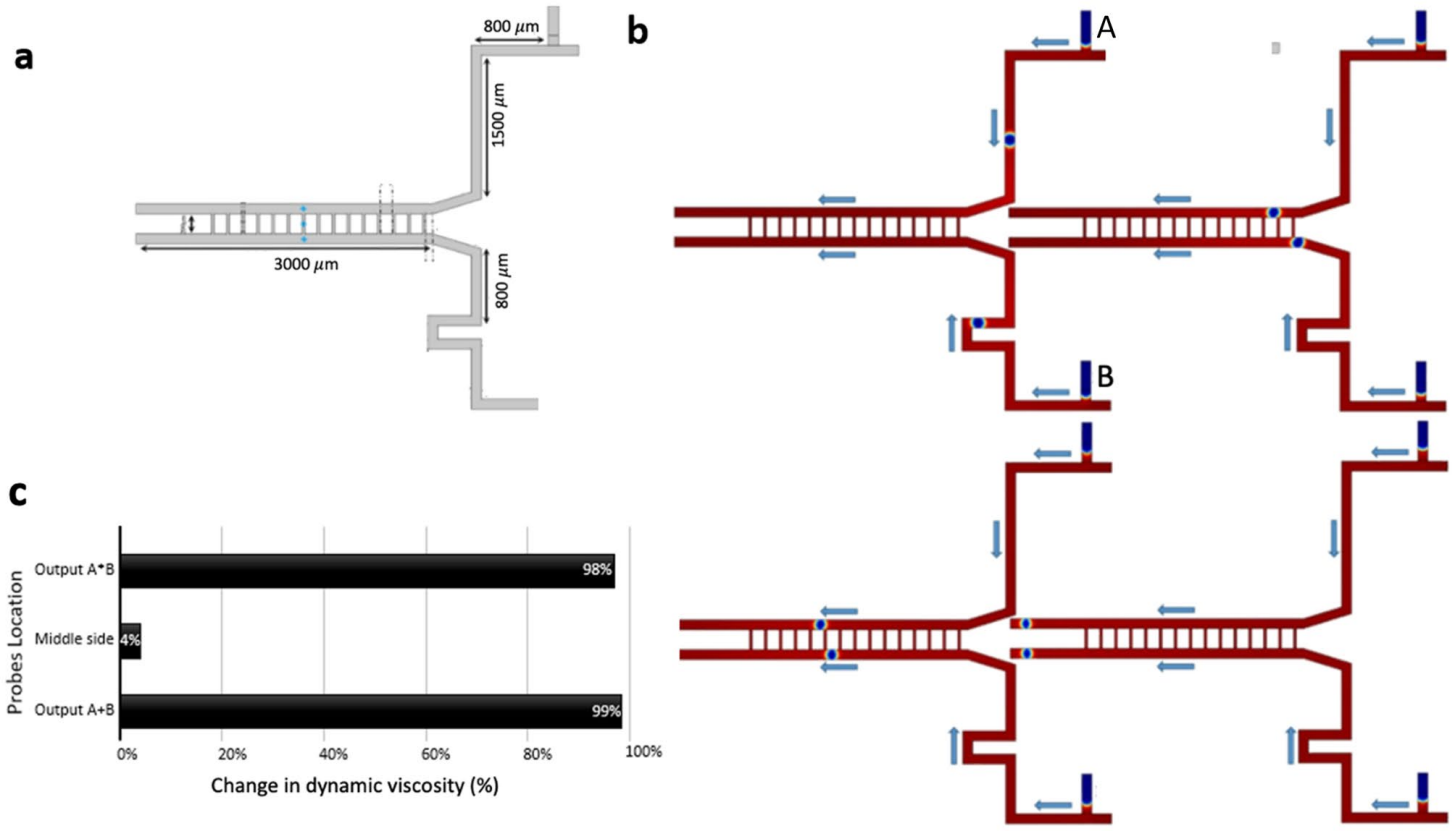
The Synchronizer microfluidic circuit and its verification. (**a**) The design and geometry of the fluidic network and probe points (blue points) of the Synchronizer. (**b**) Series of snapshots for the function of the microfluidic Synchronizer gate during one full cycle while also showing the direction of bubbles flow. (**c**) The percentage change in dynamic viscosity of three points in the upper side, middle side, and lower side. The percentage change of the dynamic viscosity in the medial side of the fluidic network is almost 0, which confirms the proper function of the Synchronizer.

For the microfluidic Synchronizer gate, the design and geometry of the Synchronizer’s microfluidic network contain two T-junctions for bubble generation (channel height = 100 µm), and with two side fluidic channels (upper and lower) connected together by 15 narrow branches placed with an equal distance from each other (Fig. 9a). The hydraulic resistance of the upper and lower fluidic channels and the connecting branches were adjusted to meet the requirement of Synchronizer for having two droplets simultaneously reaching at the outlet branches of the upper and lower sides. The main difference between the upper side and the lower side of the fluidic network is the length path that bubbles move to reach the output branches. By producing bubbles at the same time and utilizing synchronized flow rates, the design of this gate guarantees that both bubbles reach the outputs concurrently. Due to the shorter path of the upper side, the bubble produced by input $A$ reaches earlier the medial side of the channel. However, increasing the hydraulic resistance of the upper side assures that the second bubble at input *B* flows to the output earlier than the first bubble while simultaneously reaches the outlet. Similar to AND/OR gate type 2 and NOT gate type 2, to verify the validity of the computational model, the variation in dynamic viscosity was calculated at the output branches. Figure 9b shows dynamic viscosity at three probe points along the lower side of the fluidic network. Figure 9c shows the percentage change of the dynamic viscosity of the air–water mixture. As expected, the percentage change of the dynamic viscosity in the medial side of the fluidic network is almost zero due to the absence of a bubble in this region [Video S12].

### Decoder 1 to 2 microfluidic circuit

**Figure 10:**
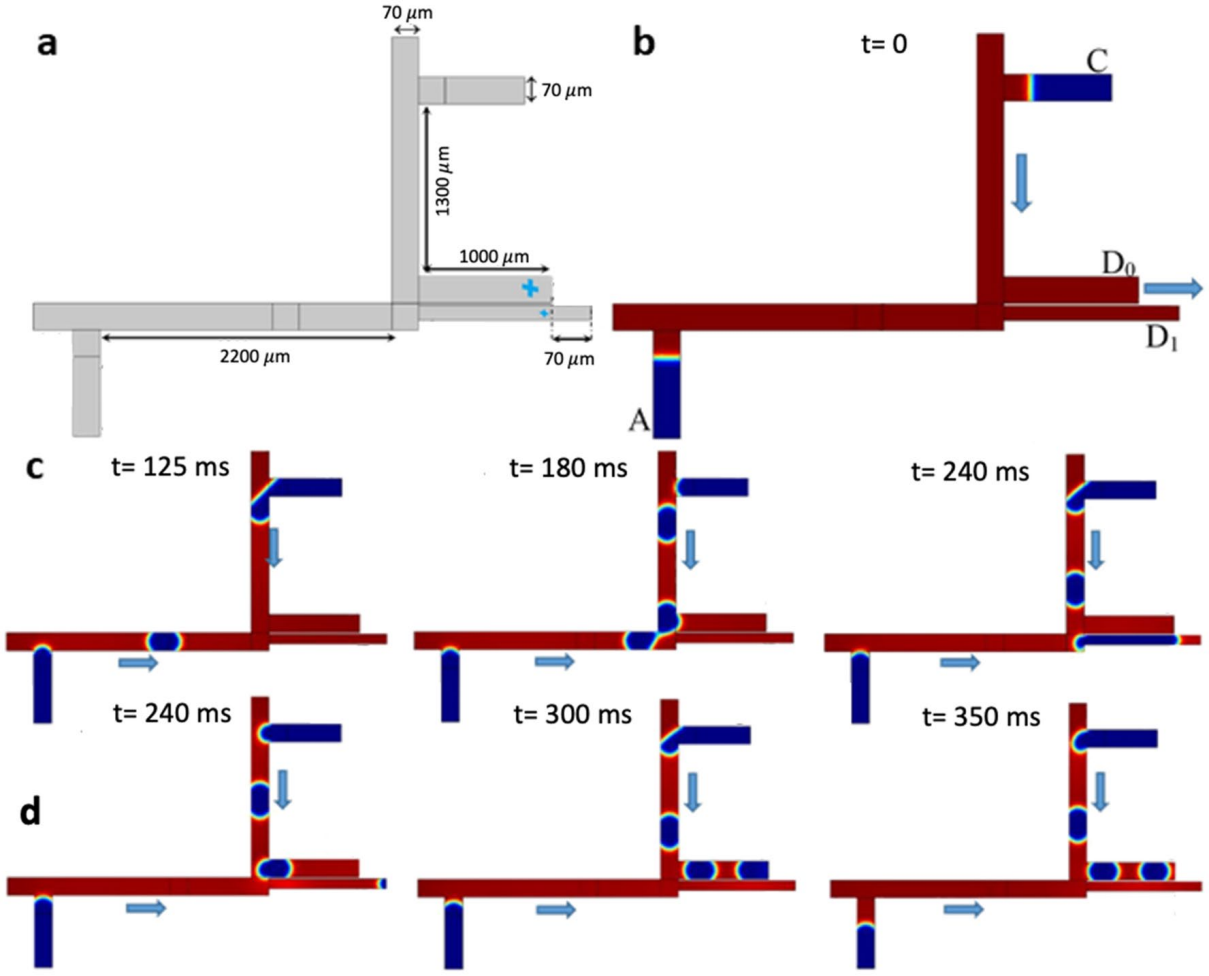
The microfluidic Decoder 1 to 2 circuit. **(a,b)** The design and geometry of the Decoder 1 to 2 circuit. **(c)** A snapshot of the case (0–1), in which only input C produces bubbles to show the asynchronous function of Decoder 1 to 2 and the direction of the bubble flow is toward the output *D*_*0*_. **(d)** Series of snapshots of the function of the case (1–1) where the direction of bubble flow is toward output *D*_*1*_.

Here we present a new heuristic design of microfluidic-based Decoder 1 to 2. The T-junctions are placed vertically and horizontally at the input branches $A$ and $C$, respectively (Fig. 10a,b). The T-junction placed horizontally is the main input *A* and produces a single bubble each time while the other one at input *C* functions as a clock and produces a train of bubbles with a certain rate of production. The design has two outputs ($D0$ and $D1$) to demonstrate the logics 0 and 1. The output branches are different from each other in length and height. Controlling of the hydrodynamic resistance of the output branches enables this microfluidic system to act as a Decoder 1 to 2. The Synchronizer gate has two logic conditions: (1) the logic condition (0–1) wherein input *A* does not produce any bubble (tagged as $A = 0$) while input $C$ that continuously produces bubbles (tagged as $C = 1$), therefore the output branches result in $D0 = 1$ and $D1 = 0$ (Fig. 10c); (2) the logic condition $(1-1)$, wherein input *A* produces a single bubble (tagged as $A = 1$) along with continuous bubble generation of bubbles via input $C$ (tagged as $C = 1$), therefore, the output branches are $D0 = 0$ and $D1 = 1$ (Fig. 10d) (Video S13 and S14).

### Decoder 2 to 4 microfluidic circuit

**Figure 11:**
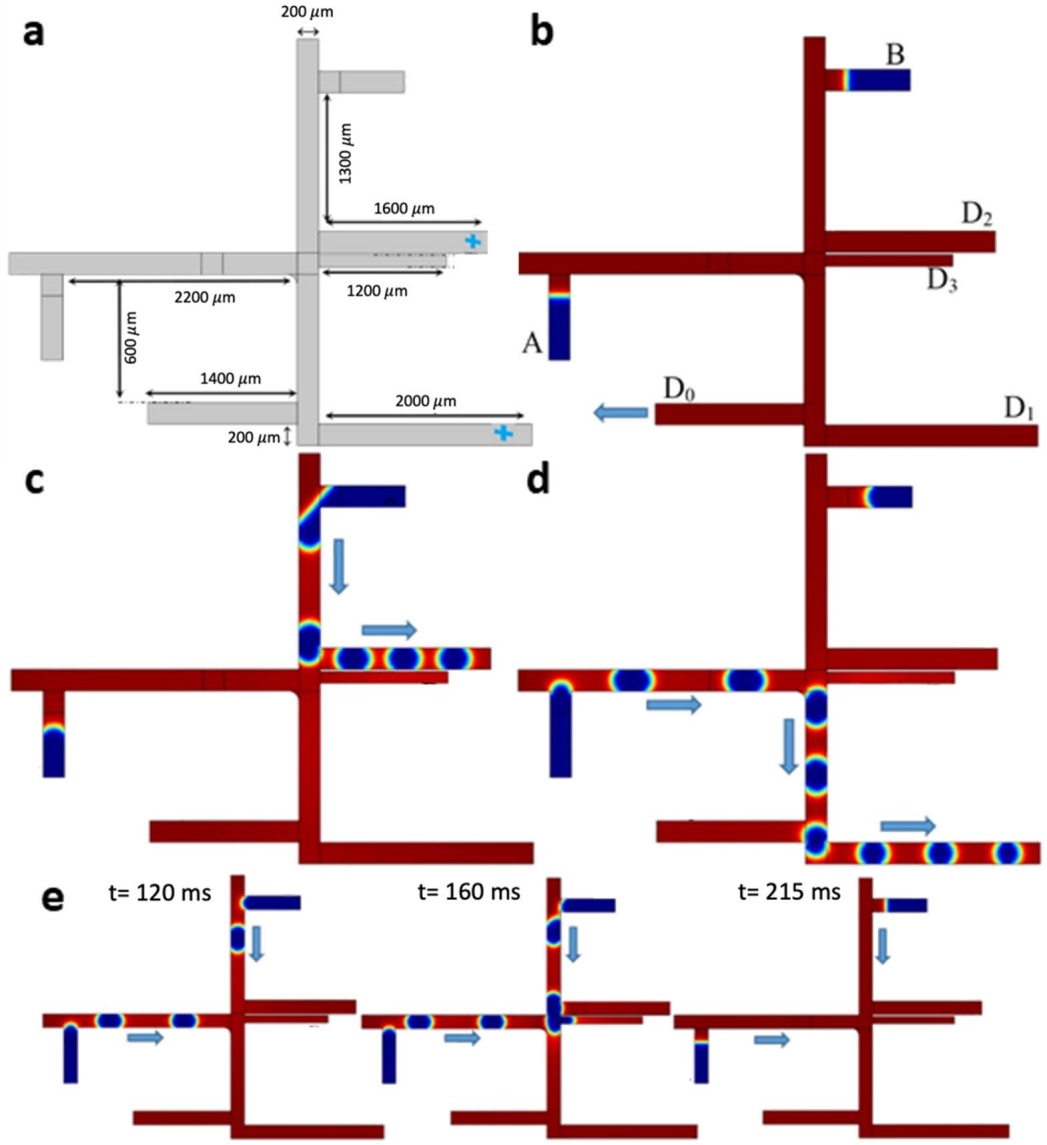
The microfluidic architecture of Decoder 2 to 4. **(a)** The design and geometry of the fluidic network for Decoder 2 to 4. **(b)** A snapshot of the logic condition $A=0, B=0$, wherein the carrier flows toward output ${D}_{0}$. **(c)** A snapshot of the logic condition $A=0, B=1$, wherein the bubbles flow toward output ${D}_{2}$. **(d)** A snapshot of the logic condition $A=1, B=0$, wherein the bubbles flow toward output ${D}_{1}$. **(e)** Series of snapshots of the logic condition $A=1, B=1$, wherein the bubbles flow toward output ${D}_{3}$.

The heuristic design of the microfluidic-based Decoder 2 to 4 is shown in Fig. 11a. This gate works in Synchronous mode. Both the inputs are considered as the primary inputs. The T-junctions are placed vertically and horizontally at the input branches $A$ and $B$ while the four outputs $D0, D1, D2$ and $D3$ are used to demonstrate the performance of the logic. The microchannel geometry and hydrodynamic resistance of the output branches are appropriately designed to act as a Decoder 2 to 4. This design has four logic conditions. In the case $A=0, B=0$, none of the bubble generators produce bubbles, therefore the output branches are zero. Although there is no bubble in the channel, we design an output gate for this condition to show that the logic gate is entirely similar to the theoretical base (Fig. 11b). In the case $A=0, B=1$, only inlet B produces bubbles and due to the Decoder 2 to 4 logic, the resulted output is $D2 = 1$ (Fig. 11c). In the case $A=1, B=0$, only inlet A produces bubbles and due to the Decoder 2 to 4 logic, the resulted output is $D1 = 1$ (Fig. 11d). It is noted that the direction of bubble flow is independent of the distance between the inputs and outputs, although it depends on the design of output branches and hydrodynamic resistance of the fluidic network. In the last scenario $A=1, B=1$, both the bubble generators produce a train of bubbles and the output branches are $D3 = 1$. The direction of bubble flow shows that the bubbles do not separate from each other when they merge at the center of the fluidic network. However, the target outlet of the merged droplets is dependent on the design of the output branches and hydrodynamic resistance of the fluidic network (Fig. 11e) (Videos S15, S16, and S17).

### Combinational circuits

**Figure 12:**
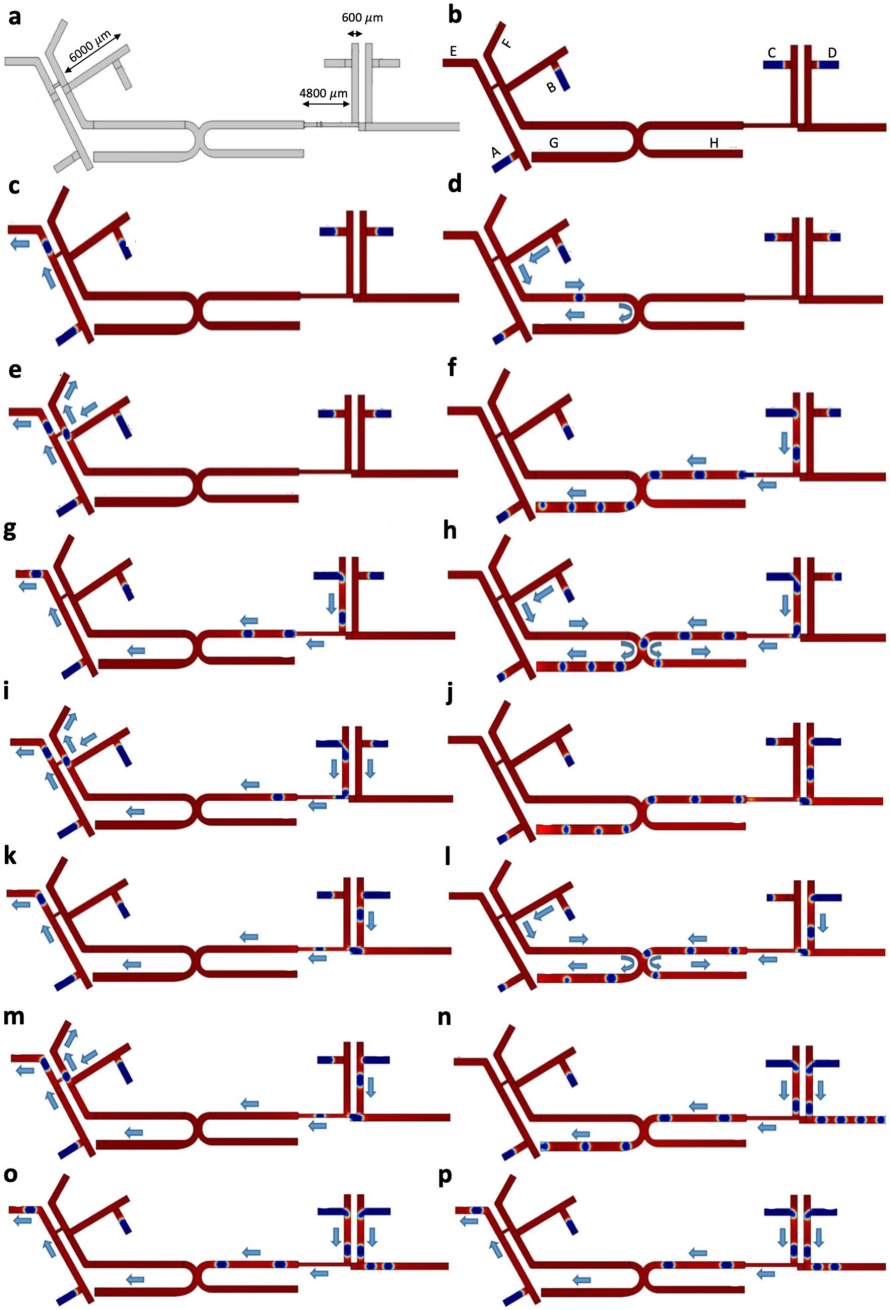
Combinational microfluidic circuit. **(a)** The design and geometry of the combinatorial circuit. **(b)** None of the bubble generators produce bubbles, and therefore there is no bubble produced at the outputs. **(c)** Input $A$ produces bubbles. Because of the NOT gate logic, the resulted output is $E=1$. **(d)** Only input $B$ produces bubbles. Because of the $(\overline{A }*B)+0$ logic, the output is $G=1$. **(e)** Both inputs $A$ and $B$ produce bubbles, and due to the NOT gate logic, the resulted outputs are $E=1$ and $F=1$. **(f)** Input $C$ produces bubbles and due to the $(C+D)+0$ logic, the output is $G=1$. **(g)** Based on cases (c) and (f), the resulted outputs are $E=1$ and $G=1$. **(h)** Inputs $B$ and $C$ produce bubbles. Because of the logics $(\overline{A }*B)+(C+D)$ and $(\overline{A }*B)*(C+D)$, the resulted outputs are $G=1$ and $H=1$. **(i)** Based on the cases (e), and (f), the resulted outputs are $E=1$, $F=1$, and $G=1$. **(j)** Input $D$ produces bubbles. Due to the $(C+D)+0$ logic, the output is $G=1$. **(k)** Based on cases (c) and (j), the resulted outputs are $E=1$ and $G=1$. **(l)** Inputs $B$ and $D$ produce bubbles, and due to the logics $(\overline{A }*B)+(C+D)$ and $(\overline{A }*B)*(C+D)$, the resulted outputs are $G=1$ and $H=1$. **(m)** Based on cases (e) and (j), the resulted outputs are $E=1$, $F=1$, and $G=1$. **(n)** Both input $C$ and $D$ produce bubbles, and due to the logics AND-OR type 2 and $(C+D)+0$, the resulted outputs are $G=1$ and $I=1$. **(o)** Based on cases (c) and (n), the resulted outputs are *G* = 1, *I* = 1 and *E* = 1. **(p)** Input $B$, $C$, and $D$ produce bubbles, and due to the logics AND-OR type 2, $(\overline{A }*B)+(C+D)$ and $(\overline{A }*B)*(C+D)$, the resulted outputs are $G=1$, $I=1$ and $H=1$. Items (b) to (p) show the cases 0 to 15 and the directions of the bubbles flow to the outputs, respectively. The detail information is shown in Table 3.

Following the development of fundamental gate elements above, here we combine these gate elements to develop a complex logical combinational circuit. Figure 12 illustrates a microfluidic circuit that combines three different logic gates NOT, AND-OR type 1, and AND-OR type 2. The proposed microfluidic circuit has four bubble generators, two first inputs for making NOT gate (inputs $A$ and $B$) and two other inputs for making AND-OR type 1 and 2 (inputs $C$ and $D$). The height of the microchannels remains constant for the entire fluidic network ($h=60 \mu m$). Figure 12a shows geometries, inputs $A$, $B$, $C$, and $D$, and outputs $E$, $F$, $G$, and $H$. The parameters used in this design are defined in Tables 1, 2. The scenarios occurring in this logical combination are presented in Table 3. In case #0, no bubble is generated in the inputs. Therefore, there is no bubble at the outputs and all the output branches result in zero (Fig. 12b). In case #1, input $A$ produces bubbles and due to the NOT gate, the resulted output is $E=1$ (Fig. 12c). In case #2, only input B produces a bubble and due to the $(\overline{A }*B)+0$ logic, the resulted output is $G=1$ (Fig. 12d). In case #3, both the inputs $A$ and B produce bubbles, and due to the NOT gate, the resulted outputs are $E=1$ and $F=1$ (Fig. 12e). In case #4, input $C$ produces bubbles, and due to the $(C+D)+0$ logic, the output $G=1$ (Fig. 12f). In case #5, based on cases #1 and #4, the resulted outputs are $E=1$ and $G=1$ (Fig. 12g). In case #6, inputs $B$ and $C$ produce bubbles and due to the logics $(\overline{A }*B)+(C+D)$ and $(\overline{A }*B)*(C+D)$, the resulted outputs are $G=1$ and $H=1$ (Fig. 12h). In case #7, based on cases #3 and #4, the resulted outputs are $E=1$, $F=1$, and $G=1$ (Fig. 12i). In case #8, only input $D$ produces bubbles, and due to the $(C+D)+0$ logic, the resulted output is $G=1$ (Fig. 12j). In case #9, based on cases #1 and #8, the resulted outputs are $E$=1 and $G=1$ (Fig. 12k). In case #10, inputs $B$ and $D$ produce bubbles and due to the logics $(\overline{A }*B)+(C+D)$ and $(\overline{A }*B)*(C+D)$, the resulted outputs are $G=1$ and $H=1$ (Fig. 12l). In case #11, based on cases #3 and #8, the resulted outputs are $E=1$, $F=1$, and $G=1$ (Fig. 12m). In case #12, both inputs $C$ and $D$ produce bubbles and due to the logics of AND-OR type 2 and $(C+D)+0$, the resulted outputs are $G$= 1, and $I=1$ (Fig. 12n). In case #13, based on cases #1 and #12, the resulted outputs are $G=1$, $I=1$, and $E=1$ (Fig. 12o). In case #14, input $B$, $C$, and $D$ produce bubbles and due to the logics of AND-OR type 2, and $(\overline{A }*B)+(C+D)$ and $(\overline{A }*B)*(C+D)$, the resulted outputs are $G=1$, $I=1$, and $H$=1 (Fig. 12p) (Video S18 and S19).

**Table 1:** Input parameters of the combinatorial microfluidic circuit.

Inputs	$\mathrm{Flow of carrier}$ ${\mathrm{phaseQ}}_{\mathrm{c}} (\mathrm{\mu l}/\mathrm{min})$	Flow of disperse phase ${\mathrm{Q}}_{\mathrm{d}}(\mathrm{\mu l}/\mathrm{min})$	$\mathrm{Dzd }(\mathrm{\mu m})$	$\mathrm{Dzd }(\mathrm{\mu m})$
Input A	114	49.8	300	120
Input B	114	49.8	300	120
Input C	114	49.8	300	200
Input D	114	49.8	300	200

**Table 2:** Output parameters of the combinatorial microfluidic circuit.

Outputs	$\mathrm{Flow rate at output}$	${\mathrm{D}}_{{\mathrm{Z}}_{\mathrm{out}}}(\mathrm{\mu m})$
Output E	$10 (\mathrm{\mu l}/\mathrm{min})$	20
Output F	$58.2 (\mathrm{\mu l}/\mathrm{min})$	771
Output H	$10 (\mathrm{\mu l}/\mathrm{min})$	32
Output I	$49.8 (\mathrm{\mu l}/\mathrm{min})$	156

**Table 3:** Logic conditions of the combinatorial microfluidic circuit for all different input cases (scenarios) and the resulted outputs.

Case	Input A	Input B	Input C	Input D	Output	${t}_{s}(\mathrm{ms})$	${t}_{stop}(\mathrm{ms})$
0	0	0	0	0	0	–	–
1	1	0	0	0	E	1	2000
2	0	1	0	0	G	1	4000
3	1	1	0	0	E and F	1	2000
4	0	0	1	0	G	1	2000
5	1	0	1	0	E and G	1	2000
6	0	1	1	0	G and H	1	4000
7	1	1	1	0	E, F and G	1	2000
8	0	0	0	1	G	1	2500
9	1	0	0	1	E and G	1	2500
10	0	1	0	1	G and H	1	4000
11	1	1	0	1	E and F and G	1	2500
12	0	0	1	1	G and I	1	2500
13	1	0	1	1	G, I and E	1	2500
14	0	1	1	1	G, I and H	1	4000
15	1	1	1	1	E, F, G and I	1	2500

## Methods

A new set of passive microfluidic-based logic gates were developed to implement various microfluidic circuit processors. A Multiphysics computational analysis was recruited to endorse the behavior of bubbles (droplets) flowing in microfluidic networks and simulate various logic gates. The phase-field method was used in a 2D model to decrease the simulation time while still preserving the precision of the models. Overall, eight different logic gates in addition to a parametric T-junction as a bubble producer were modeled in this work. We also designed a microfluidic circuit that is a combination of multiple gates, involving AND/OR type 1, AND/OR type 2, and NOT. The CPU time needed for simulating each of the gates (by a personal computer Asus-FX553V-Intel Core i7-7700HQ 2.8 GHz-16 GB RAM, 64 bits) is reported in Table 4.

**Table 4:** The CPU time required for simulating the microfluidic-based logic gate models of water–air droplet microfluidic systems developed in this work.

Logic gate	t_CPU_ (min)	Logic gate	t_CPU_ (min)
AND-OR type 1, case (1–1)	65	Decoder 2 to 4, case (0–0)	0
AND-OR type 1, case (1–0)	63	Circuit, case 0	0
AND-OR type 1, case (0–1)	116	Circuit, case 1	24
AND-OR type 2, case (1–1)	7	Circuit, case 2	50
AND-OR type 1, case (1–0)	7	Circuit, case 3	64
AND-OR type 1, case (0–1)	7	Circuit, case 4	100
NOT type 1, case (1–1)	32	Circuit, case 5	100
NOT type 1, case (0–1)	30	Circuit, case 6	120
NOT type 2, case (1–1)	9	Circuit, case 7	110
NOT type 2, case (0–1)	9	Circuit, case 8	110
Flip-Flop	44	Circuit, case 9	110
Synchronizer	32	Circuit, case 10	145
Decoder 1 to 2, case (1–1)	13	Circuit, case 11	135
Decoder 1 to 2, case (0–1)	11	Circuit, case 12	180
Decoder 2 to 4, case (1–1)	27	Circuit, case 13	180
Decoder 2 to 4, case (1–0)	50	Circuit, case 14	265
Decoder 2 to 4, case (0–1)	20	Circuit, case 15	200

### Digital fluidic logical gate

**Figure 13:**
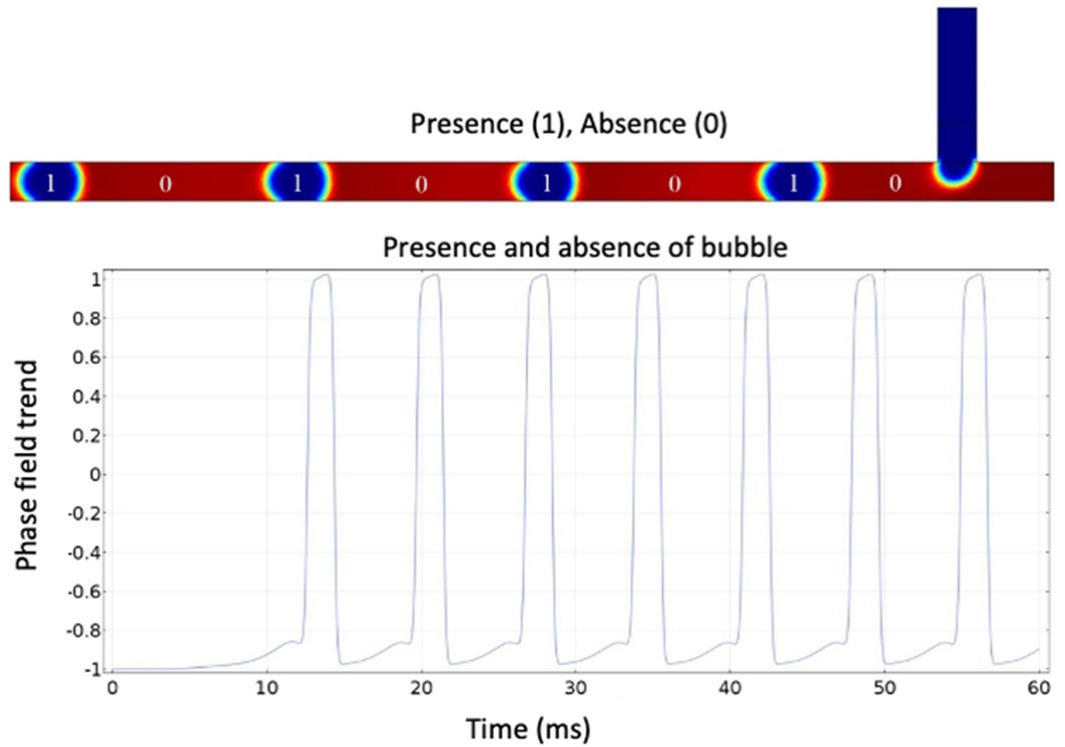
The presence or absence of bubbles in a microchannel and its corresponding phase-field trend. Each bubble represents a bit of data, wherein the presence of bubble = 1 and absence of bubble = 0. The phase-field trend shows the presence of a bubble when it reaches the probe point (output port).

The dispersed phase transporting within the carrier phase represents a discrete bubble (droplet) and creates a signal by specifying the presence or absence of the bubble as a binary of data (logical 1 or logical 0). Figure 13 illustrates the streaming of microfluidic-based bit data. This intrinsic property of the droplets in a microchannel is used to produce microfluidic-based logic processors.

### Parameters assumptions

COMSOL Multiphysics operating system was employed to solve the Computation Fluid Dynamic (CFD) models. Several parameters were considered constant in all simulations unless otherwise mentioned. These constant parameters are testing temperature ($T=20^\circ{\rm C}$), atmospheric pressure ($P=1 atm$), density and dynamic viscosity of the air ($18.27 \times {10}^{-6} \mu \left(Pa . s\right)$ and 1*.*225 $\rho \left(\frac{Kg}{m3}\right)$, respectively), and density and dynamic viscosity of the water ($1.95 \times {10}^{-3 }\mu (Pa . s)$ and ${10}^{3}\rho (\frac{Kg}{m3}),$ respectively). The water and air are selected as the two insoluble fluids in distinct phases in this study. The air is the carrier phase, and the water is the dispersed phase. The surface tension ($\sigma$) between the water and air is set to 0.072 ($N/m$)^15,22^.

### Definition of heuristic microfluidic gates and related designing parameters

Six different logic gates, including two types of AND-OR and NOT gates, a Flip-flop (memory), a synchronizer, and a T-junction model were simulated in this work. In addition, two other gates called: Decoder 1 to 2 and Decoder 2 to 4 were designed and simulated in the synchronous and asynchronous modes. Finally, one microfluidic circuit that combines three of the individual gates was introduced and simulated in one new circuit. The definition of the essential parameters related to these microfluidic models are presented in Table 5. $h$ is microchannel height, *D* is longest path between the input and output gate of the microfluidic network, *t*_*s*_ is time step, *t*_*stop*_ is end time of the simulation, ${D}_{{z}_{c}}$ is entrance length of the carrier phase, ${D}_{{z}_{d}}$ is entrance length of the dispersed phase and ${D}_{{z}_{out}}$ is entrance length of the outputs.

**Table 5:** The parameters required for simulating the logic gates.

Logic gate	${\mathrm{Q}}_{\mathrm{c}} (\mathrm{\mu l}/\mathrm{min})$	${\mathrm{Q}}_{\mathrm{d }}(\mathrm{\mu l}/\mathrm{min})$	$\mathrm{h }(\mathrm{\mu m})$	$\mathrm{D }(\mathrm{mm})$	$\mathrm{ts }(\mathrm{ms})$	$\mathrm{tstop }(\mathrm{ms})$	$\mathrm{hc }(\mathrm{\mu m})$	$\mathrm{hd }(\mathrm{\mu m})$
T-junction	$10.02$	$10.02$	100	1.2	${10}^{-1}$	20	40	70
AND-OR type 1	$10.02$	$10.02$	50	1.54	${110}^{-1}$	75	200	200
AND-OR type 2	$114$	$49.8$	100	14	1	650	700	600
NOT type 1	$49.8$	$58.2$	70	1.1	1	80	400	420
NOT type 2	$49.8$	$58.2$	100	1.95	1	130	200	200
Flip-Flop	$33.6$	$48.6$	70	1.4	1 110	13.5	69	69
Synchronizer	$10.02$	$8.94$	100	4.3	1	225	50	200
Decoder 1 to 2	$49.8$	$114$	20	2.6	${110}^{-1}$	220	300	1000
Decoder 2 to 4	$49.8$	$114$	20	4.4	1	190	300	1000

Two types of input gates are considered in our circuit designs: $A=0$ or $B=0$ which means $Qc = Qd = 0$; and $A=1$ or $B=1$ (Tables 5, 6).

**Table 6:** Output flow rate and entrance length for each output branch.

Outputs	Q_out_	${\mathrm{D}}_{{\mathrm{z}}_{\mathrm{out}}} (\mathrm{\mu m})$
AND/OR type 1, output $A+B$	$0 (\mathrm{Pa})$	–
AND/OR type 1, output $A * B$	$1.67\times {10}^{-9}({\mathrm{m}}^{3}/\mathrm{s})$	200
AND/OR type 2, output $A+B$	$0 (\mathrm{Pa})$	–
AND/OR type 2, output $A * B$	$8.3\times {10}^{-10}({\mathrm{m}}^{3}/\mathrm{s})$	415
NOT type 1, output $A$	$0 (\mathrm{Pa})$	–
NOT type 1, output $A * B$	$9.7\times {10}^{-10}({\mathrm{m}}^{3}/\mathrm{s})$	700
NOT type 1, output $\overline{A }* B$	$9.7\times {10}^{-10}({\mathrm{m}}^{3}/\mathrm{s})$	830
NOT type 2, output $\overline{A }$	$0 (\mathrm{Pa})$	–
NOT type 2, output ${D}_{1}$	$9.7\times {10}^{-10}({\mathrm{m}}^{3}/\mathrm{s})$	200
NOT type 2, output ${D}_{2}$	$9.7\times {10}^{-10}({\mathrm{m}}^{3}/\mathrm{s})$	540
Flip-Flop	$0 (\mathrm{Pa})$	–
Synchronizer	$0 (\mathrm{Pa})$	–
Decoder 1 to 2, output *D*0	$0 (\mathrm{Pa})$	–
Decoder 1 to 2, output *D*1	$8.3\times {10}^{-10}({\mathrm{m}}^{3}/\mathrm{s})$	170
Decoder 2 to 4, output *D*0	$0 ({\mathrm{m}}^{3}/\mathrm{s})$	1
Decoder 2 to 4, output *D*1	$8.3\times {10}^{-10}({\mathrm{m}}^{3}/\mathrm{s})$	300
Decoder 2 to 4, output *D*2	$8.3\times {10}^{-10}({\mathrm{m}}^{3}/\mathrm{s})$	400
Decoder 2 to 4, output *D*3	$0 (\mathrm{Pa})$	–

Table 7 illustrates the simulation detail of each logic gate, wherein ${t}_{in}$ is average time the first bubble forms, $tout$ is average time the first bubble reaches the outlet, $L$ is bubble length, and $\Delta$ is average distance between two bubbles.

**Table 7:** Efficiency and meshing element status for each logical gate. ${t}_{in}$ is average time the first bubble forms, ${t}_{out}$ is average time the first bubble reaches the outlet, and $L$ is bubble length.

Logic gate	$\mathrm{tin }(\mathrm{ms})$	$\mathrm{tout }(\mathrm{ms})$	$\mathrm{L }(\mathrm{\mu m})$	Mesh element size
AND-OR type 1	10.9	65	220	Fine
AND-OR type 2	157	230	360	Fine
NOT type 1	12	57	180	Finer
NOT type 2	10	120	180	Fine
Flip-Flop	2.3	8.5	250	Finer
Synchronizer	50	225	140	Extra fine
Decoder 1 to 2	65	221	40	Finer
Decoder 2 to 4	67	190	40	Finer

## Supplementary Information

Supplementary Information.

Supplementary Video 1.

Supplementary Video 2.

Supplementary Video 3.

Supplementary Video 4.

Supplementary Video 5.

Supplementary Video 6.

Supplementary Video 7.

Supplementary Video 8.

Supplementary Video 9.

Supplementary Video 10.

Supplementary Video 11.

Supplementary Video 12.

Supplementary Video 13.

Supplementary Video 14.

Supplementary Video 15.

Supplementary Video 16.

Supplementary Video 17.

Supplementary Video 18.

Supplementary Video 19.

Supplementary Legends.
