# Supplementary material for: Microfluidic-based processors and circuits design
Source: Sci Rep. 2021 May 26;11:10985. doi: 10.1038/s41598-021-90485-z (PMC8155008; doi:10.1038/s41598-021-90485-z)
Supplement: Supplementary file 1 — Supplementary Information. [file 41598_2021_90485_MOESM1_ESM.docx]

Microfluidic-Based Processors and Circuits Design

Kasra Azizbeigi^1^, Maysam Zamani Pedram^1, 2, 3^ and Amir Sanati-Nezhad ^2, 3^

^1^ Faculty of Electrical Engineering, K.N. Toosi University of Technology, Tehran, Iran

^2^ Department of Mechanical and Manufacturing Engineering, University of Calgary, Calgary, Alberta T2N 1N4, Canada

^3^ Center for Bioengineering Research and Education, Biomedical Engineering Program, University of Calgary, Calgary, Alberta, T2N 1N4, Canada

**Supplemental Information**

**S1. The design of the proposed T-Junction microfluidic structure**

***S1.1. Channel height***

Referring to **Figure 2**, the geometries were designed in a parametric format in which all the dimensions are scaled to and presented with a factor of channel height (h).

***S1.2. Input and output flow rates***

The input flow rates of the carrier and dispersed phases were set to $10.02 {\mu l}/s$ which ensures a reasonable correlation between the computational time and real testing conditions. In the T-junction model, the flow rate of both the carrier and dispersed fluids $(Qc,Qd )$ were set equal: $Qc = Qd = 10.02 {\mu l}/s$. The output pressure was set to atmospheric pressure. The flow of the carrier phase (air flow) occurs when the flow rate or the entrance length of the dispersed phase is equal to zero and the value of the phase-field trend is approximately equal to $-1$. The flow of the droplets initiates when the entrance length of the dispersed phase is slightly greater than the entrance length of the carrier phase, where its phase field trend fluctuates between $-1$ and $+1$. The Slug-plug flow occurs when the entrance length of the dispersed phase is slightly smaller than the entrance length of the carrier fluid. The stratified flow occurs when the entrance length of the dispersed phase is much smaller than the entrance length of the carrier phase. The dispersed phase flow (water flow) occurs when the flow rate or entrance length of the carrier phase is equal to zero, where the phase-field trend is approximately equal to $+1$. Bubbles produced are proportionally larger at increasing the entrance length. These reactions are consistent with the experimental results reported somewhere else.^1^

***S1.3. Contact angle***

Always with the empirical situations, the bubble wets the wall even if it crosses the slim layer of the carrier phase. To explore surface wetting, water/air droplet simulations are performed in contact angle range $\theta\in[155^{\circ}, 55^{\circ}]$. The simulation results show that bubbles were reliably formed at the T junction at the contact angle of about$135^{\circ}$.

| 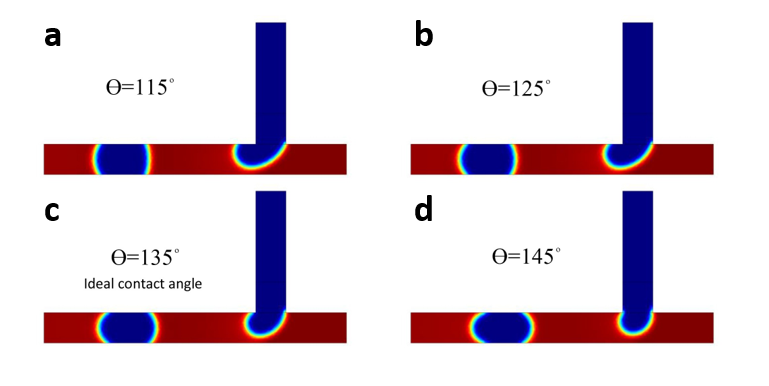 |
| --- |
| **Figure S1.** Different contact angles for shaping the bubbles in the T-junction water/air model. To represent our simulation as close as possible to real experimental microfluidic logic gates, the value of $135^{\circ}$ was selected for the contact angle between the water bubble and the microchannel walls. |

**Table S1.** Three different degrees of meshing used in the T-junction numerical model.

| **Mesh degree** | **Number of mesh elements** | $\boldsymbol{<\Delta> (\mu}\boldsymbol{m}\mathbf{)}$ | *t_CPU_* (min) |
| --- | --- | --- | --- |
| Coarse | 223 | 22.75 | 0.75 |
| Normal | 798 | 11.75 | 4.75 |
| Fine | 1844 | 7.1 | 88 |

***S1.4. Mathematical modeling and phase-field method***

Two-phase flow operations are modeled by coupling the Navier-Stokes (NS) equation and the Phase-Field (PF) method.^2^ The phase-field method is based on fluid free energy and suggests an alluring supersede to the better-settled techniques to solve multiphase flow models. Other than tracking the interface between the two fluid phases, an interface layer is administered by a phase field variable ($\phi$). The surface tension body force with a gradient ($\phi$) is added to the Navier-Stokes equation. The theoretical equations in this study include continuity equation and the Navier-Stokes equation combined with surface tension ($G\nabla\phi$), gravity ($g$), pressure tensor ($\nabla P$), the Cahn-Hilliard equation to sample the Intersection dynamics, and the chemical potential $G$ representing the change in the levels of free energy as below.

| $\nabla\cdot v = 0$ | (S1) |
| --- | --- |

| $\rho\left( \frac{\partial v}{\partial t}+v.\nabla v \right)=-\nabla P+\rho g+\nabla\mu\left( \nabla v+\left( \nabla v \right)^{T} \right)+G\nabla\phi$ | (S2) |
| --- | --- |

| $\frac{\partial\phi}{\partial t}+v.\nabla\phi=\gamma\nabla^{2}G$ | (S3) |
| --- | --- |

The Cahn-Hilliard (CH) equation includes 4^th^-order derivative making it more complicated than the NS equation to solve.

| $G=\lambda(-\nabla^{2}\phi+\frac{\phi\left( \phi^{2}-1 \right)}{\varepsilon^{2}})$ | (S4) |
| --- | --- |

The mobility parameter is a diffusion parameter ($\gamma$) in equation S5. The capillary width ($\varepsilon$) controls the thickness of the intersection. The mobility tuning parameter is $\chi$ and the density of the interfacial energy is $\lambda$ in equation S6.

| $\gamma=\chi\varepsilon^{2}$ | (S5) |
| --- | --- |
| $\lambda=\frac{3\varepsilon\sigma}{2\sqrt{2}}$ | (S6) |

The phase-field variable $\phi$ ranges between $-1$ and $+1$. Also, it has the values of $\pm1$ in two separated phases; $\phi=+1$ for the gas phase (dispersed phase) and $\phi=-1$ for the liquid phase (carrier phase). Furthermore, the fluid interface is the area between these two phases. Therefore, the viscosity $\mu$ and density $\rho$ rely upon the phase field.

| $\rho=\frac{1-\phi}{2}\rho_{1}+\frac{1+\phi}{2}\rho_{2}$ | (S7) |
| --- | --- |
| $\mu=\frac{1-\phi}{2}\mu_{1}+\frac{1+\phi}{2}\mu_{2}$ | (S8) |

The resolution of the interface thickness is key in phase-field methods. It is expected to have the leastwise four cells wide interface for a precise performance for calculating two-phase flows on a stable mesh. However, the phase-field method is permitted to have an interface resolution only up to two cells wide.^3^ To affirm the mesh-independence, different estimations of $\varepsilon$ and $\gamma$ have been examined for the T-junction model.

**S2. Phase-field effect and efficiency of logic gates**

Considering the phase-field variable as the parameter to analyze the efficiency of logic gates, the presence or absence of bubbles is shown as values 1 and 0, respectively. In other words, the presence of the dispersed phase is set as value 1, and the presence of the carrier phase is set as value 0 in the microchannel. It is noted that the dynamics of the fluid flow in microchannels in the simulation is dependent on the quality of meshing (**Figure S**2).

| 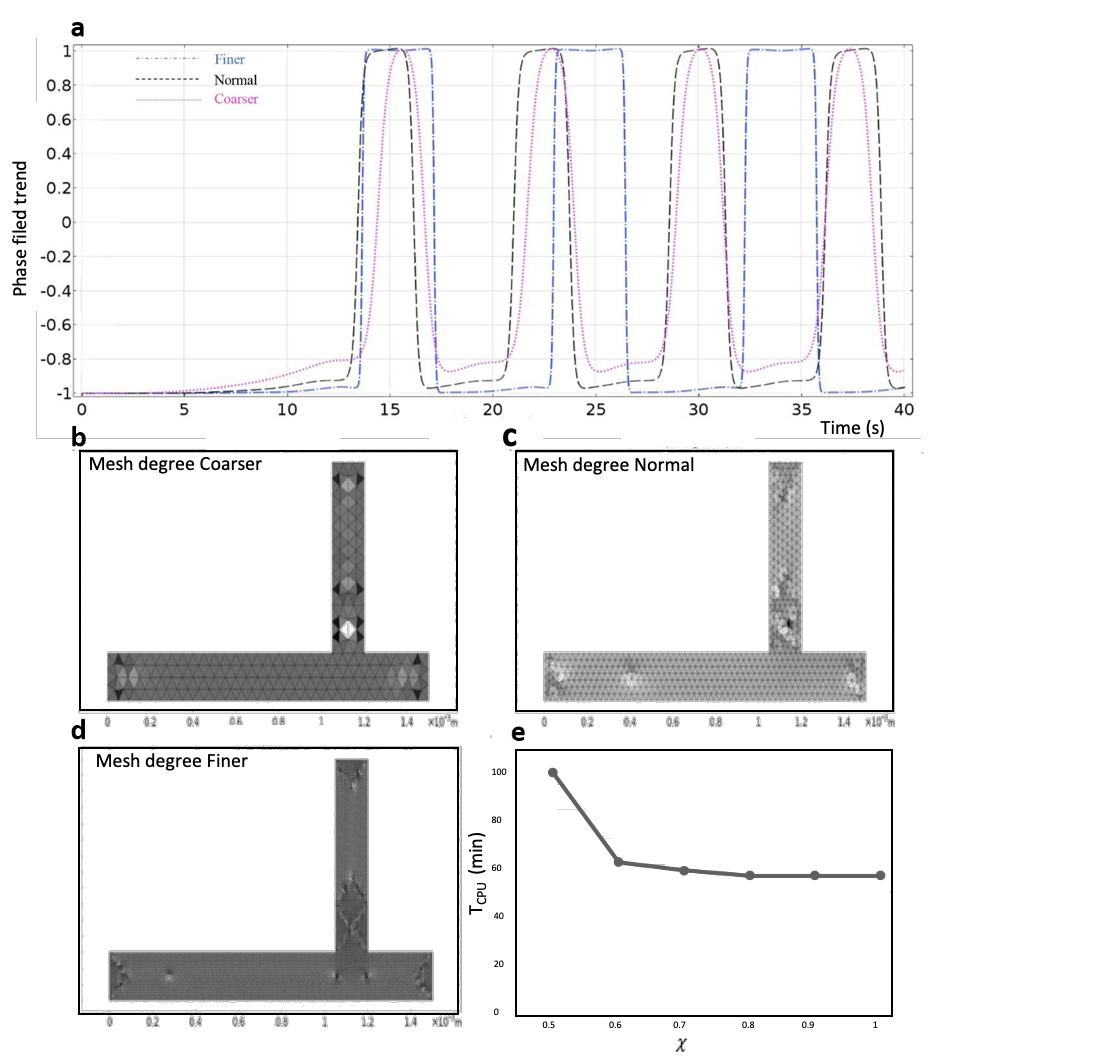 |
| --- |
| **Figure S2. Phase-field effect on logic gates. (a)** Phase-field trends of the three meshes for the T-junction model. **(b-d)** Coarse, normal and fine degrees of the meshing. **(e)** Mobility curves for the T-junction ($\varepsilon=0.5$). |

**References**

1 Duffy, D., McDonald, J., Schueller, O. & Whitesides, G. Rapid prototyping of microfluidic systems in poly(dimethylsiloxane). *Analytical Chemistry* **70**, 4974-4984, (1998).

2 Zhou, C., Yue, P. & Feng, J. Deformation of a compound drop through a contraction in a pressure-driven pipe flow. *International Journal of Multiphase Flow* **34**, 102-109, (2008).

3 Worner, M. Numerical modeling of multiphase flows in microfluidics and micro process engineering: a review of methods and applications. *Microfluidics and Nanofluidics* **12**, 841-886, (2012).
