# Supplementary material for: Microfluidic-based processors and circuits design
Source: Sci Rep. 2021 May 26;11:10985. doi: 10.1038/s41598-021-90485-z (PMC8155008; doi:10.1038/s41598-021-90485-z)
Supplement: Supplementary file 21 — Supplementary Legends. [file 41598_2021_90485_MOESM21_ESM.docx]

**Supplementary Video Legends**

**Supplementary Video 1**. The video of microfluidic circuit AND/OR type 1, state 1

**Supplementary Video 2**. The video of microfluidic circuit AND/OR type 1, state 2

**Supplementary Video 3**. The video of microfluidic circuit AND/OR type 1, state 3

**Supplementary Video 4**. The video of microfluidic circuit AND/OR type 2, state 1

**Supplementary Video 5**. The video of microfluidic circuit AND/OR type 2, state 2

**Supplementary Video 6**. The video of microfluidic circuit AND/OR type 2, state 3

**Supplementary Video 7**. The video of microfluidic circuit NOT type 1, state A

**Supplementary Video 8**. The video of microfluidic circuit NOT type 1, state B

**Supplementary Video 9**. The video of microfluidic circuit NOT type 2, state A

**Supplementary Video 10**. The video of microfluidic circuit NOT type 2, state B

**Supplementary Video 11**. The video of microfluidic Flip Flop circuit

**Supplementary Video 12**. The video of microfluidic Synchronizer circuit

**Supplementary Video 13**. The video of microfluidic circuit Decoder 1 to 2, state 1

**Supplementary Video 14**. The video of microfluidic circuit Decoder 1 to 2, state 2

**Supplementary Video 15**. The video of microfluidic circuit Decoder 2 to 4, state 1

**Supplementary Video 16**. The video of microfluidic circuit Decoder 2 to 4, state 2

**Supplementary Video 17**. The video of microfluidic circuit Decoder 2 to 4, state 3

**Supplementary Video 18**. The microfluidic combinational circuit, state 1

**Supplementary Video 19**. The microfluidic combinational circuit, state 2
